# Challenges and Promise for the Development of Human Immune Monitoring

**DOI:** 10.5041/RMMJ.10090

**Published:** 2012-10-31

**Authors:** Shai S. Shen-Orr

**Affiliations:** Department of Immunology, Rappaport Institute of Medical Research, Bruce Rappaport Faculty of Medicine, Technion – Israel Institute of Technology, Haifa, Israel

**Keywords:** Genomics, immune monitoring, immunology, immunosenescence, mass cytometry, systems immunology

## Abstract

The immune system is critical for protection and health maintenance and is likely required for a long lifespan. Yet, despite its importance for health, the ability to assess its quality of function has been poor, nor is much known on its variation between individuals. Hence direct assessment of immune health has largely been missing from medicine, and metrics of immune health are not well defined, especially in non-extreme states. This is chiefly due to the high complexity of the immune system. Recently emerging technologies now enable broad surveying of many immune system components at high resolution, setting forth a transformation of immunology and, through it, medicine. Such technologies enable, for the first time, high-resolution monitoring of an individual’s immune system. The resulting information can be used for diagnostic and prognostic purposes, as well as to provide a quantitative, global view of the immune system, i.e. “systems immunology.” This is especially relevant in the context of aging, in which the immune system exhibits profound alterations in state and function.

## INTRODUCTION

Immunity to infectious diseases is orchestrated by a highly complex system of specialized cells and organs that flourishes on diversity and is in a constant interplay with its environment. Today, roughly 50 years since the inception of modern immunology, the immune system is considered a rich and complex system whose basic mechanisms are largely understood. As in other fields, a reductionist approach has been the predominant research strategy in immunology research for many years. Immunologists working in various subfields have made huge advances toward understanding particular segments of the immune system. Medical interventions have arisen from this basic science work, beginning with vaccines for infectious diseases, to the more recent development of monoclonal antibodies used to treat a variety of diseases. The total impact of immunological interventions, vaccinations in particular, on human health has been great, especially when considering molecular-based health interventions.

Despite these medical successes and a good understanding of basic mechanisms of immunity, it appears as though we have only scratched the surface. As technological breakthroughs have enabled ever more sophisticated research tools, additional layers of complexity have been revealed. For example, new immune cell subtypes (i.e. cells with differing functionality at a given condition) have been discovered such that the estimates for the total number of distinct cell subtypes now number in the hundreds. As in other large complex systems, cooperativity and cross-talk abound in the immune system, and these likely play a key role, as protective immunity is ultimately an emergent phenomenon whose functionality is greater than the sum of its parts. A view with this richness in mind would suggest that our understanding of how the immune system functions as a whole is very limited.

**Figure 1:**
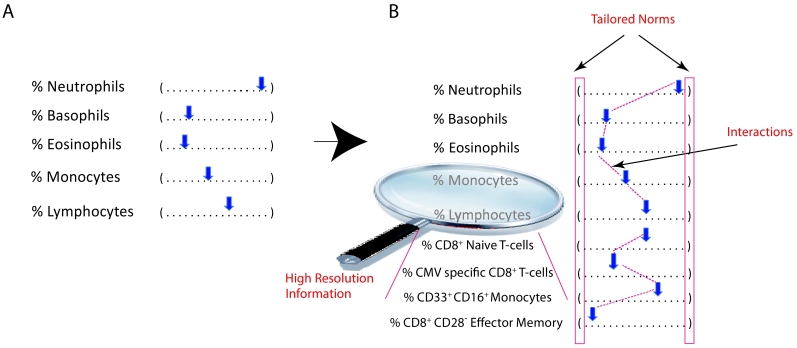
**The standard complete blood count (CBC) test provides little information on immune function.** **(A)** Immune cell subset information in a standard CBC is scant and has changed little over the past 60 years, despite increased knowledge of immune function. **(B)** Future standard tests for enumerating immune cell subsets should include increased resolution covering those cell subsets found to be clinically actionable, tailored normal ranges fit for an individual’s background (e.g. age, ethnicity, etc.), and explicit quantification of parameter combinations whose interactions are important, difficult for clinicians to detect otherwise, especially as the number of parameters quantified grows, yet reflect a clinically informative global immune state. The frequency in blood of the additional cell subsets appearing in the example “future CBC” have all been shown to alter significantly with age. (CMV, cytomegalovirus).

At the clinical level, despite its importance for general health, many aspects of the immune system are mostly ignored, and little is known about the variation in immune system components and their functions. The standard “complete blood count” (CBC), one of the most commonly prescribed tests by physicians, is indicative of a recent infection or extreme disease cases such as drastic reduction in cell counts (Figure 1A). First used clinically in 1957, the test enumerates the five major leukocyte classes in blood based on cell shape and size (later automated versions of this assay replaced shape with electrical impedance). Yet enumeration of the many immune cell subtypes, discovered since and identified through the specific expression of protein markers, is not achievable through a CBC test. In specific cases, flow cytometry is used clinically for enumerating additional cell subsets. Yet such profiling is performed only in specific disease cases to confirm a disease association (e.g. TH17 cell dysfunction in autoimmunity), or to monitor immune reconstitution (e.g. B, CD4+/CD8+ T cells ratio in bone-marrow transplant) rather than prospectively for prevention or early detection. Moreover, such clinical immunology testing assays are performed at a cell subset resolution far below the complexity known to exist in the immune system, yielding partial results inferred over heterogeneous cell types. With such scant collected data, the clinical implication of fluctuations of immune cell subsets among either healthy or diseased individuals is not known, nor is the relationship between subsets quantified or normal ranges tailored to an individual’s background. Hence, there is room for an improved substitution CBC assay (Figure 1B). With more comprehensive data, there is an improved chance of predicting a person’s response to vaccination, drug treatments, disease susceptibility, or outcome; all these processes, in which the immune system likely plays a critical role, are currently beyond the reach of clinical immunology and medicine at large.

This paper describes recent technological and methodological advances in measuring immune system function. In stark contrast to other critical organ systems, such as the cardiovascular system, the question “Doc, how is my immune system doing?” has no good answer at present, especially for those individuals with no cases of extreme disease. The new advances in measuring the immune system may for the first time provide deeper understanding of the role of immunity in health and the development of immune health metrics.

## MEASURING IMMUNITY CAN NOW BE PERFORMED COMPREHENSIVELY AND AT A HIGH RESOLUTION

### High-Resolution Cell Subset Enumeration by Mass Cytometry

Cells are the quanta of the immune system, and their identity and function can be understood by the degree to which they express proteins on the cell surface or intracellularly. Traditionally, the work-horse tool of immunology has been the flow cytometer, which optically measures the return fluorescence from cells stained with fluorophore-labeled antibodies bound to proteins. It is through functional characterization of cells expressing specific combinations of proteins that much of our understanding of immune system functionality has been gained and the many different cell types, each with their own functionality, have been delineated. Thus, a drive towards being able to measure an increasing number of fluorophores on single cells was present from the early days of flow cytometry development.^1^ Yet a difficulty for using multiple fluorophores simultaneously in flow cytometry is that the fluorescence emission spectrum of one fluorophore “spills over” from its characteristic wave length to interfere with readings from other fluorophores. This strongly reduces the ability to accurately gauge the abundance of each protein in the cell. Thus, at expert flow cytometry centers, with substantial effort one can use up to 10–15 different fluorophores before the overlapping emission spectra become too complex to be accurately separated, whereas for most flow cytometry centers, let alone clinically based ones, it would be a struggle to accurately measure even that many labels together.

**Figure 2:**
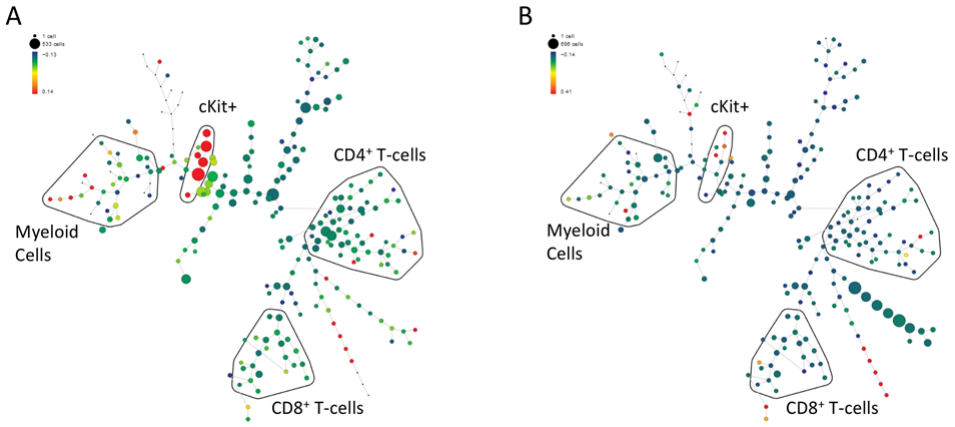
**Mass cytometry captures a snapshot of immune system alterations in cell subset abundance between conditions.** Immunophenotypic differences between human bone-marrow samples of a cancer patient taken **(A)** pre- and **(B)** post-chemotherapy, measured by mass cytometry using antibodies against 25 cell surface antigens and analyzed and visualized using the SPADE algorithm in a tree plot.4 Antigen abundances define each individual cell measured as a single point in a 25-dimensional space. Individual cells most similar to one another are clustered into one of 200 clusters (nodes) connected by similarity to form a tree-like structure. The size of each node in the tree corresponds to the number of cells that fall into the node, whereas the color is scaled to the median intensity of marker expression of the cells within each node (cKit is shown). Putative cell populations were annotated manually and are represented by colored lines encircling sets of nodes that have CD marker expression emblematic of the indicated subset designations. Significant changes in cell subsets pre- and post-chemotherapy are observed both in the drastic reduction in cell subset population sizes (compare size of individual nodes between **A** and **B**) and intensity of cKit expression (compare color between **A** and **B**).

Mass cytometry is a recently introduced technology (the commercial product is called a “CyTOF” for cytometry by time-of-flight; DVS Sciences, Mountain View, CA, USA) that measures the abundance of heavy metal isotope labels on antibodies and other tags (such as peptide-MHC tetramers for labeling specific T cells) on single cells using mass spectroscopy.^2^,^3^ Unlike flow cytometry, which is grounded in the physics of optics, the advantage of mass cytometry is that no optical equivalent of “spill-over” exists in mass, such that many more molecules may be used in combination to assay a single sample (blood or single cell suspension of tissues).^4^–^7^ Currently, isotopic-label antibody panels spanning over 35 proteins are already run regularly via mass cytometry (110 different proteins is the upper limit), and much more information may be obtained from each cell. An illustration of the revolution in scale this transformative technology provides is that while 10 labels may yield approximately 1,000 possible combinations of cell subsets, 30 yield 1,000,000 (using the formula *X* = 2^*n*−1^, where *X* is the number of combinations and *n* is the number of labels). Of note, repeated runs of a flow cytometer on the same sample, each time measuring a different combination of antibodies, will not have equivalent coverage to the mass cytometer as the data will not be obtained on the same single cell. Thus there is much greater resolving power with the number of labels possible with this new technology, enabling simultaneous quantification of the majority of known immune cell types from an individual sample, including rare cell subsets (Figure 2).

### Functional Signaling Assays at the Single Cell Level

For primary tissue, original applications of flow cytometry allowed only the quantification of cell surface markers. However, developments over the past decade, primarily in the lab of Garry Nolan at Stanford University, have enabled the quantification of intracellular proteins, and of particular interest, phosphorylated epitopes.^8^,^9^ This allows for accurate quantification at the single cell level of phospho-signaling biochemistry (phospho-flow). Performed comparatively for a sample between an unstimulated and stimulated condition, the resultant difference or fold-change is interpreted as the degree of activation of the specific signaling pathways probed.^9^,^10^ With mass cytometry, the strength of this approach has grown even further as it allows multi-parameterization at an even greater scale including both cell type and functional response to stimulation by cell surface intracellular markers respectively.^4^,^5^,^11^,^12^ Thus, the machinery and systems now exist to access the heterogeneity of cellular subsets in the immune system and enable phenotypic characterization and functional assays at a resolution previously unavailable to immunologists.

### Multiplex Measures of Serum Proteins

The immune system is very much dependent on the interactions of various white blood cells with each other, either in synaptic contacts, or at a distance using secreted proteins such as cytokines or chemokines for regulation and response. Cytokine levels in body fluids, such as serum or cerebrospinal fluid, can be quantitated, and due to their importance for immunity their measurement has always been of interest. Yet, the family of cytokines, chemokines, and more generally secreted factors involved in immunity is large, including many redundancies in its effects on cellular response and regulation, with many cytokines exhibiting multiple functionality that is context-dependent. Until recently only a handful of cytokines could be measured simultaneously in a study, and the resultant partial capture of this complex milieu did not yield high clinical utility.

In recent years, several technological platforms enabling simultaneous measurement of serum protein in multiplex have become available. Most popular amongst them are the bead array multiplex assays based on the Luminex technology (Luminex Corporation, Austin, TX, USA). These can measure up to 500 analytes from very small blood volumes, for almost 100 samples at a time. Kits geared for immune-phenotyping of up to 51 serum proteins in one assay well are already available from several vendors. Hence, it is a new day for attempts to identify predictive signatures of disease associations from body fluid-detectable proteins.

### TCR and BCR Repertoire Analysis through Next-Generation Sequencing

The adaptive arm of immunity tailors responses for any encountered antigen. To do so, B and T cells generate an enormous repertoire of structural diversity in antigen-recognizing proteins, including antibodies and T cell receptors (TCR), through a gene segment rearrangement process which combines variable, diverse, and joining gene segments, known as VDJ recombination. An allelic exclusion mechanism generally allows only a single VDJ combination to be expressed in a given cell, despite the additional chromosomal copies, and a separate mechanism-activated post-antigen recognition assures high specificity of the receptor/antibody to the antigen through hyper-mutation and selection. As many as 10^8^ different combinations can be created by VDJ recombination, and repertoire diversity is thought to be critical for protective immunity. With an estimated cell count of 10^11^ different B and T cells in an individual human being, it is assumed that this mechanism generates a sufficiently large repertoire for immune system antigen recognition. However, until recently, surveying even a small fraction of an individual’s repertoire was considered an impossible task.

Next-generation DNA sequencing now offers the opportunity of starting to explore the basic principles of repertoire selection as well as its relation to disease. Through the design of primers flanking regions of interest, in-depth sequencing of a representative sampling of repertoire diversity may be achieved. First studies performing deep sequencing of antibody and TCR sequences have all reported that the VDJ recombination is biased.^13^–^15^ That is, it does not occur with equal probability for each combination. Moreover, large numbers of distinct combinations are expressed in a single individual at a single point in time^15^ (over 10^6^ for TCR in humans, though still negligible compared with the upper theoretical limit of 10^11^), and a significant overlap of sequence repertoires between any two individuals can be observed.^16^ Similarly, a high-bandwidth low-cost deep sequencing approach has recently been described for sequencing human leukocyte antigen (HLA) regions.^17^ The size, diversity, and affinity of this repertoire is expected to be closely linked with immune response. Hence, exploring this diversity and its clinical implications in an individual or a population is of high importance.

### Cell Type-Specific and Single Cell Gene Expression Assays

Much of our knowledge in immunology comes from bulk measurements of many cells together. Due to problems of averaging and noise, the behavior of cells as inferred from average measurements often drowns cell-to-cell differences and may not reflect the behavior of any single cell.^18^ Beyond measuring a select few biological species across many single cells and different cell types (e.g. in flow or mass-based cytometry), measurement of many biological species (e.g. whole genome gene expression) has been dictated by cost limitations, technological hurdles, and the difficulty of analyzing *en masse* single cell data. Hence, it is often the case that multiple cell types are analyzed as a single tissue, such as happens for example when gene expression is analyzed in bulk from whole blood, and the resulting analysis to a large degree describes the heterogeneity of the tissue, rather than the underlying biological changes in condition that are of interest.

Recent methodological and technological innovations now elevate some of these difficulties and enable the in-depth exploration of several biological species in many cell types or single cells. These include computational methodologies to infer cell type-specific information from heterogeneous tissue data,^19^,^20^ microfluidic devices used to measure and image cells run in multiplex (across multiple cells and genes),^21^,^22^ and, emerging now, single cell whole genome measures.^23^,^24^ These methodologies and technological innovations have enabled the measurement of single cell cytokine secretion^25^ and cell counting from minute samples,^26^,^27^ to name but a few. The miniaturization of these devices and their relative low cost and transportability are promising for the future development of microfluidics-based diagnostics. The sensitivity of cell type-specific measures performed through these techniques often offers orders of magnitude higher resolution than that obtained by analyzing heterogeneous measures of tissue and cells and reveal novel biological phenomena masked by cell-to-cell or cell type-to-cell type variation.

## INTEGRATIVE ANALYSIS OF THE IMMUNE SYSTEM IN A ONE-STOP SHOP

In a highly interconnected system such as immunity, it would be expected that changes in one component of the immune “network” will affect other connected components.^28^,^29^ A methodology which focuses on measuring only a few variables, as traditionally has been applied in immunology, will not be able to relate the changes observed in one variable to measurement of others. This applies specifically to any single study as well as across the entire immune literature. For example, several studies in the elderly have reported reduced lymphocyte proliferation to new antigens,^30^,^31^ and others have reported an increased number of CD8+ T cells lacking the co-stimulatory molecule CD28;^32^–^34^ but would they be observed in any one individual? The technologies discussed above enable a high-bandwidth (though not yet comprehensive) enumeration of immune system components and their abundance at the cell subset, serum protein, gene expression, or sequence level, providing the first answers to these questions.

**Figure 3:**
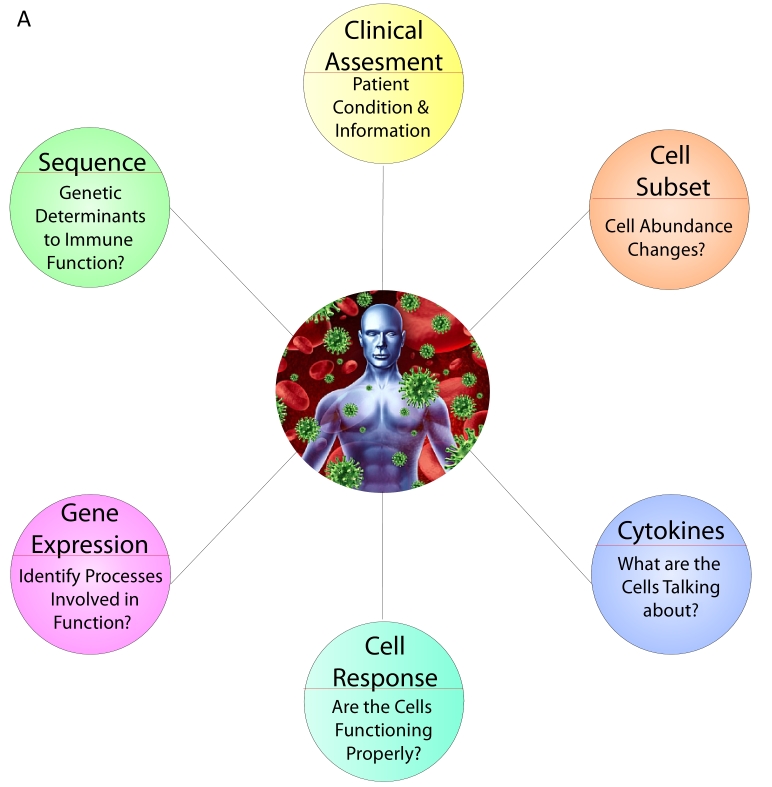

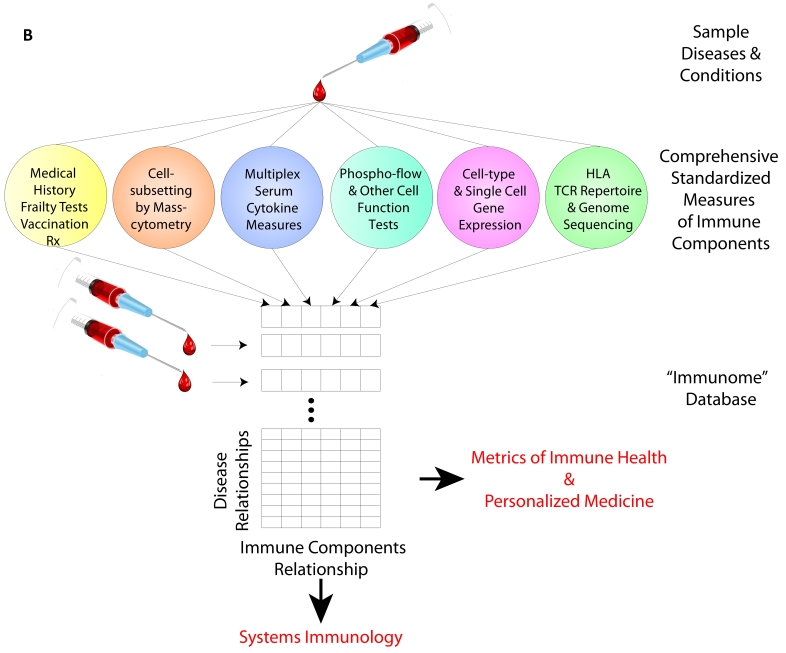
**A model for one-stop shop human immune monitoring and a standardized, hospital-driven, immunome project.** **(A)** Transformative technological advances now enable measurement of multiple parts of the immune system at high bandwidth. Measurement of multiple such modalities from a single sample and their integrated analysis can yield a detailed view of an individual’s immune state which may be informative for clinical decisions. **(B)** High-resolution analysis of multiple diseases and conditions in a standardized one-stop shop manner allows comparisons across samples and between immune compartments. This will be a bedrock for building a systems-level understanding of the immune system and spear-head a new direction for personalized medicine, more informative than genetic analysis alone.

At present, the high-bandwidth technologies available and discussed here measure distinct components of the immune system: cells types, their communication with one another, functionality, and specificity. Although these parts are rich in novel information, a more sophisticated level of analysis would integrate multiple components to glean a full view of immunity in man (Figure 3A). The interconnected nature of the immune system would suggest that one layer strongly affects another, yet at this stage it is not clear to what extent measures of one layer would be informative towards another. For instance, to what degree can one estimate serum protein measures from the abundance of measured gene expression for gene coding that protein, or learn about cell subset frequencies from measured gene expression data,^20^ cell signaling from cell subsets, or cell signaling response from the serum protein which stimulate them? Initial findings from our lab and those of others suggest that the different components of the immune system do indeed reflect what is going on in other parts of the system, but that the reflected information is only partial and a full picture cannot be gleaned without surveying additional components.

From these findings, a profiling methodology arises which calls for one-stop shop immune monitoring.^35^ That is, comprehensive measurement of multiple parts of the immune system from a single sample. Such profiling, deployed now in an increasing number of “immune monitoring centers” around the world, ourselves included, is yielding massive amounts of data on the immune system of a single individual (Figure 3B).^36^ Powerful information may be gained through the use of standardized sample assays and shared data repositories that will allow sample comparisons across diseases and experiments. Paralleling the grand scale nature of the Human Genome Project, a call for a large scale “Immunome” project has been made, with the purpose of assaying the diversity of the human immune system in health and disease and establishing proper metrics of immune health.^35^ Though the financial burden of such measurements is high, the data are extremely rich and reveal interactions between the different components of the immune system. Such data enable, for the first time, formulation of a quantitative, system-level view of immunity. With such knowledge, the hope is to be able to identify comprehensively not only all components of an individual’s immune system, but a more narrow set of measurements (likely spanning multiple immune components) from which predictive metrics of immune health may be defined resulting in the actualization of clinical personalized medicine (Figure 3B).

## THE CLINICAL BENEFITS OF INCREASED RESOLUTION OF IMMUNE FUNCTION

Historically, the ability to dissect biological phenomena with increased resolution has been closely tied not only to new discovery but to increased understanding of disease heterogeneity leading to improved detection and treatment outcomes. For medicine, the above-described technological innovations will primarily be judged by their ability to deliver clinically actionable information for improved diagnosis and treatment. The leap in resolution these technologies provide for each of the parts of the immune system surveyed is orders of magnitude higher than any technological or methodological progress to date. This is revealing striking variation even between antigen-specific single cells previously thought to be identical.^5^ It may be the case, and likely for the first time in immunology, that we have reached a level of measurement accuracy that can capture the noise of the immune system itself. How the immune system handles noise to produce a robust response is likely a fascinating basic research question, but one less likely to be of clinical relevance, as such fluctuations are handled naturally by the system itself. If so, as in other fields of biology, delineating the natural noise from that which shows important functional differences would be of high relevance. Until then, the extent to which this spectacular resolution will be clinically actionable remains to be determined.

Some of the first published studies using these advanced technologies suggest that clinically valuable information may be learned from increased resolution. The direct relevance of insights gained varies by assay type, the analyses performed with the generated data, and the appropriateness of the assay for probing the disease studied. Particularly strong evidence for clinical relevance has come from the results of phospho-flow, a technique first applied close to a decade ago. Here studies have illustrated an ability to identify hyper-responsive cell subsets negatively prognostic of tumor progression,^37^ perform disease sub-classification based on signaling aberrations corresponding to clinical correlates,^38^ and understand drug mechanisms.^9^,^39^ With the arrival of mass cytometry, the power of phospho-flow analysis increases greatly as multiple signaling pathways can be profiled simultaneously and in all cells of the immune system. First examples of mass cytometry measurements of human samples have highlighted the strong ability of the technology to identify the response of cell subsets to therapy^4^ (Figure 2), bone-marrow development, as well as cell subset specificity to drugs.^11^

In gene expression, novel methodologies for analyzing heterogeneous tissue microarray data to yield cell type-specific expression have been used on whole blood samples from pediatric renal transplant patients to identify monocyte-specific differences between acute rejection and stable patients, undetectable otherwise,^19^ and for detection of expression correlates with cancer,^40^ to name but two examples. The more recent techniques for analyzing single cell gene expression data have been shown to identify novel CD8+ T cell subsets with immunization-specific gene expression signatures in human samples,^41^ and it would be expected that single cell techniques will grow in their clinical utility especially for such fields as gynecology and hematology. Serum protein and TCR repertoire analysis is still in the early days; however, results are already promising: distinctive serum protein profiles are being identified for diseases, especially auto-immune diseases, yet their predictive ability is still an open question. In contrast, repertoire sequencing of healthy individuals and blood cancer patients has already been shown to identify disease-specific signatures allowing determination of the number and identity of the dominant cancerous B cell clonal receptors.^42^ Thus, while still in the early days, the evidence suggests that high-resolution immune monitoring is scientifically justified in the context of investigating human disease. As discoveries continue, the use in the clinics will likely be driven by costs and the ability to identify an economic number of robustly measured variables that are predictive towards a specific disease or condition or more generally to our immune health.

## THE RELEVANCE OF IMMUNE MONITORING TO AGING AND IMMUNOSENESCENCE

The chances of an elderly individual contracting an infectious disease and developing complications are significantly higher than those of a younger person. Protective vaccination of these older adults is less than half as effective as that of younger individuals.^43^ The principal reason for this is thought to be a loss of immune function, termed immune senescence. For example, it has been shown that older adults can exhibit a number of immune deficiencies, in both the innate and adaptive arms of immunity including reduced lymphocyte proliferation to new antigens (both B and T cells), failure to produce neutralizing antibodies, as well as changes in the frequency of white blood cell subtypes,^44^,^45^ altered hematopoiesis, and reduced T cell receptor repertoire and antibody production.^46^

Due to the complexity of the immune system, studies of immunosenescence published to date have focused on different aspects of immunological function, and an understanding of how the different alterations observed in immunity with aging has been missing. Moreover, there are unresolved disagreements in the literature, such as whether IL-6 levels in blood increase with age.^47^ This is likely due to the high variability observed amongst older adults, which may be due to confounding co-morbidities, environmental effects, or temporal accumulation of changes. This would suggest that aging and immune aging in particular should be treated not as a single “natural disease,” but rather as either different phenotypic subsets or perhaps even to the degree of an individual personalized level. Indeed, a subpopulation of older adults has already been identified to possess an “immune risk profile,” which is associated with an age above 85, persistent cytomegalovirus infection, loss of the CD28 co-stimulatory molecule on CD8+ T cells, and increased chances of mortality in longitudinal studies.^48^ Thus it seems likely that a functional immune system is important to good health and a long lifespan.

Indeed, gene expression studies of longevity in humans, comparing tissues from young and older adults, commonly identify an age association or differential expression of many immune-related genes. However, due to increased disease and inflammation with age, tissue from older adults often contains a higher proportion of lymphocytes, making the reliable identification of age-associated immune system genes and pathways difficult.^49^ From an evolutionary perspective, the selective pressure on lifespan is thought to cease not long after the age of reproduction. Thus the immune system may have evolved for short-term effect rather than optimum protection throughout an organism’s lifespan. To date, the relationship between longevity and immunosenescence has not been explored in depth. Poor health in old age may be due to loss of immune responsiveness, whereas extended lifespan may be associated with retaining the functionality of the immune system.

The critical nature of the immune system for health and the high variation observed among older adults would suggest that differences in immune system parameters (immune states) between individuals have a significant role in determining disease susceptibility and outcome. The logic here is simple: The immune system is known to be important in a large variety of diseases and is suspected to be involved in many more. Thus if variation exists in the immune system, especially if that variation is large and is temporally stable, one would expect it to have an effect on the health of an individual and how he/she combats disease. High-resolution immune monitoring, as described here, appears to be perfectly poised to address the complexity of the immune system in general and the high variability observed in the elderly in particular. The strength comes from the comprehensive and integrated manner in which each individual is profiled and the high resolution each assay provides, coupled to standard clinical information. This enables us to observe within single individuals the relationship between multiple factors and in effect spear-heads a new direction for personalized medicine, one that is much closer to health and disease than mere genetic information.

## Abbreviations:

CBC: complete blood count;
CyTOF: cytometry by time-of-flight;
HLA: human leukocyte antigen;
TCR: T cell receptors;
VDJ: variable, diverse, and joining.

## References

[1] Tung JW, Heydari K, Tirouvanziam R, Sahaf B, Parks DR, Herzenberg LA (2007). Modern flow cytometry: a practical approach. Clin Lab Med.

[2] Ornatsky O, Bandura D, Baranov V, Nitz M, Winnik MA, Tanner S (2010). Highly multiparametric analysis by mass cytometry. J Immunol Methods.

[3] Ornatsky O, Baranov VI, Bandura DR, Tanner SD, Dick J (2006). Multiple cellular antigen detection by ICP-MS. J Immunol Methods.

[4] Bendall SC, Simonds EF, Qiu P (2011). Single-cell mass cytometry of differential immune and drug responses across a human hematopoietic continuum. Science.

[5] Newell EW, Sigal N, Bendall SC, Nolan GP, Davis MM (2012). Cytometry by time-of-flight shows combinatorial cytokine expression and virus-specific cell niches within a continuum of CD8+ T Cell phenotypes. Immunity.

[6] Ornatsky OI, Kinach R, Bandura DR (2008). Development of analytical methods for multiplex bio-assay with inductively coupled plasma mass spectrometry. J Anal At Spectrom.

[7] Bandura DR, Baranov VI, Ornatsky OI (2009). Mass cytometry: technique for real time single cell multitarget immunoassay based on inductively coupled plasma time-of-flight mass spectrometry. Anal Chem.

[8] Krutzik PO, Trejo A, Schulz KR, Nolan GP (2011). Phospho flow cytometry methods for the analysis of kinase signaling in cell lines and primary human blood samples. Methods Mol Biol.

[9] Schulz KR, Danna EA, Krutzik PO, Nolan GP (2007). Single-cell phospho-protein analysis by flow cytometry. Curr Protoc Immunol.

[10] Perez OD, Nolan GP (2006). Phospho-proteomic immune analysis by flow cytometry: from mechanism to translational medicine at the single-cell level. Immunol Rev.

[11] Bodenmiller B, Zunder ER, Finck R (2012). Multiplexed mass cytometry profiling of cellular states perturbed by small-molecule regulators. Nat Biotechnol.

[12] Behbehani GK, Bendall SC, Clutter MR, Fantl WJ, Nolan GP (2012). Single-cell mass cytometry adapted to measurements of the cell cycle. Cytometry A.

[13] Weinstein JA, Jiang N, White RA, Fisher DS, Quake SR (2009). High-throughput sequencing of the zebrafish antibody repertoire. Science.

[14] Jiang N, Weinstein JA, Penland L, White RA, Fisher DS, Quake SR (2011). Determinism and stochasticity during maturation of the zebrafish antibody repertoire. Proc Natl Acad Sci U S A.

[15] Robins HS, Campregher PV, Srivastava SK (2009). Comprehensive assessment of T-cell receptor beta-chain diversity in alphabeta T cells. Blood.

[16] Robins HS, Srivastava SK, Campregher PV (2010). Overlap and effective size of the human CD8+ T cell receptor repertoire. Sci Transl Med.

[17] Wang C, Krishnakumar S, Wilhelmy J (2012). High-throughput, high-fidelity HLA genotyping with deep sequencing. Proc Natl Acad Sci U S A.

[18] Altschuler SJ, Wu LF (2010). Cellular heterogeneity: do differences make a difference?. Cell.

[19] Shen-Orr SS, Tibshirani R, Khatri P (2010). Cell type-specific gene expression differences in complex tissues. Nat Methods.

[20] Abbas AR, Wolslegel K, Seshasayee D, Modrusan Z, Clark HF (2009). Deconvolution of blood microarray data identifies cellular activation patterns in systemic lupus erythematosus. PLoS One.

[21] Sanchez-Freire V, Ebert AD, Kalisky T, Quake SR, Wu JC (2012). Microfluidic single-cell real-time PCR for comparative analysis of gene expression patterns. Nat Protoc.

[22] Kalisky T, Blainey P, Quake SR (2011). Genomic analysis at the single-cell level. Annu Rev Genet.

[23] Tang F, Lao K, Surani MA (2011). Development and applications of single-cell transcriptome analysis. Nat Methods.

[24] Tang F, Barbacioru C, Wang Y (2009). mRNA-Seq whole-transcriptome analysis of a single cell. Nat Methods.

[25] Han Q, Bradshaw EM, Nilsson B, Hafler DA, Love JC (2010). Multidimensional analysis of the frequencies and rates of cytokine secretion from single cells by quantitative microengraving. Lab Chip.

[26] Garcia D, Ghansah I, Leblanc J, Butte MJ (2012). Counting cells with a low-cost integrated microfluidics-waveguide sensor. Biomicrofluidics.

[27] Leblanc J, Mueller AJ, Prinz A, Butte MJ (2012). Optical planar waveguide for cell counting. Appl Phys Lett.

[28] Barabasi AL, Gulbahce N, Loscalzo J (2011). Network medicine: a network-based approach to human disease. Nat Rev Genet.

[29] Schadt EE, Bjorkegren JL (2012). NEW: network-enabled wisdom in biology, medicine, and health care. Sci Transl Med.

[30] Effros RB (2000). Long-term immunological memory against viruses. Mech Ageing Dev.

[31] Remarque EJ (1999). Influenza vaccination in elderly people. Exp Gerontol.

[32] Schmidt D, Goronzy JJ, Weyand CM (1996). CD4+ CD7-CD28-T cells are expanded in rheumatoid arthritis and are characterized by autoreactivity. J Clin Invest.

[33] Goronzy JJ, Fulbright JW, Crowson CS, Poland GA, O’Fallon WM, Weyand CM (2001). Value of immunological markers in predicting responsiveness to influenza vaccination in elderly individuals. J Virol.

[34] Saurwein-Teissl M, Lung TL, Marx F (2002). Lack of antibody production following immunization in old age: association with CD8(+)CD28(−) T cell clonal expansions and an imbalance in the production of Th1 and Th2 cytokines. J Immunol.

[35] Davis MM (2008). A prescription for human immunology. Immunity.

[36] Human Immunology Project Consortium (HIPC) (2012). http://www.immuneprofiling.org.

[37] Irish JM, Myklebust JH, Alizadeh AA (2010). B-cell signaling networks reveal a negative prognostic human lymphoma cell subset that emerges during tumor progression. Proc Natl Acad Sci U S A.

[38] Kotecha N, Flores NJ, Irish JM (2008). Single-cell profiling identifies aberrant STAT5 activation in myeloid malignancies with specific clinical and biologic correlates. Cancer Cell.

[39] Bendall SC, Nolan GP, Roederer M, Chattopadhyay PK (2012). A deep profiler’s guide to cytometry. Trends Immunol.

[40] Stuart RO, Wachsman W, Berry CC (2004). In silico dissection of cell-type-associated patterns of gene expression in prostate cancer. Proc Natl Acad Sci U S A.

[41] Flatz L, Roychoudhuri R, Honda M (2011). Single-cell gene-expression profiling reveals qualitatively distinct CD8 T cells elicited by different gene-based vaccines. Proc Natl Acad Sci U S A.

[42] Boyd SD, Marshall EL, Merker JD (2009). Measurement and clinical monitoring of human lymphocyte clonality by massively parallel VDJ pyrosequencing. Sci Transl Med.

[43] Goodwin K, Viboud C, Simonsen L (2006). Antibody response to influenza vaccination in the elderly: a quantitative review. Vaccine.

[44] Longo DL, Paul WE (2003). Immunology of Aging. Fundamental Immunology.

[45] Miller RA (1996). The aging immune system: primer and prospectus. Science.

[46] Weiskopf D, Weinberger B, Grubeck-Loebenstein B (2009). The aging of the immune system. Transpl Int.

[47] Beharka AA, Meydani M, Wu D, Leka LS, Meydani A, Meydani SN (2001). Interleukin-6 production does not increase with age. J Gerontol A Biol Sci Med Sci.

[48] Strindhall J, Nilsson BO, Lofgren S (2007). No immune risk profile among individuals who reach 100 years of age: findings from the Swedish NONA immune longitudinal study. Exp Gerontol.

[49] Rodwell GE, Sonu R, Zahn JM (2004). A transcriptional profile of aging in the human kidney. PLoS Biol.

